# From Kigali to Rio: advancing an evidence‐based and equitable HIV response

**DOI:** 10.1002/jia2.70064

**Published:** 2025-11-27

**Authors:** Beatriz Grinsztejn, Richard M. Ochanda, Sara M. Allinder, Rena Janamnuaysook, Andrew Grulich, Kenneth Ngure

**Affiliations:** ^1^ Fundação Oswaldo Cruz–Instituto Nacional de Infectologia Evandro Chagas Rio de Janeiro Brazil; ^2^ AVAC Nairobi Kenya; ^3^ Center for Innovation in Global Health Georgetown University Washington DC USA; ^4^ Institute of HIV Research and Innovation Bangkok Thailand; ^5^ University of New South Wales Sydney New South Wales Australia; ^6^ School of Public Health Jomo Kenyatta University of Agriculture and Technology Juja Kenya

1

From the earliest days of the HIV epidemic, communities of people living with, and affected by, HIV have mobilized to advocate, driving political action, transforming research and service delivery, and fighting for equitable access to lifesaving care. This leadership has enabled millions to survive and thrive. Over the past year, the global HIV response has entered a period of heightened uncertainty, marked by significant funding cuts, widening inequities in prevention and treatment access, and growing pressure on health systems to sustain progress with fewer resources. As funding declines, human rights have been eroded, and science and global health assistance have become politicized, placing progress at risk. Meeting this moment requires renewed commitment to the values that have defined the HIV response: science, solidarity and social justice.

At IAS 2025 in Rwanda, over 700 scientists, advocates and public health leaders adopted the Kigali Declaration, a call to protect and build on more than four decades of progress against a disease that has killed over 44 million people [1, 2]. The declaration outlined five principles: embracing meaningful partnerships; supporting global HIV research; prioritizing HIV prevention; protecting human rights; and rejecting the politicization of science.

Building on this momentum, IAS—the International AIDS Society, through its *Road to Rio* initiative, launched a global consultation to accelerate implementation of these principles ahead of AIDS 2026—the 26th International AIDS Conference that will take place in Rio de Janeiro, Brazil. Participants identified three key advocacy priorities rooted in science and community leadership: sustaining investment in HIV science; ensuring that health systems are person‐centred and grounded in human rights; and securing equitable access to prevention to achieve epidemic control.

These messages form a framework for coordinated action to advance priorities through a unified, evidence‐based approach.

**Investment in science saves lives**

Continued investment in HIV research will drive the next generation of breakthroughs, benefiting public health far beyond the HIV field. The same research that revolutionized HIV care has already transformed multiple medical disciplines, including chimeric antigen receptor T‐cell therapy for cancer, curative hepatitis C treatments [3], and insights into cardiovascular, autoimmune and blood conditions [4]. The development of HIV research infrastructure and implementation capacity have enabled faster, more coordinated responses to other health challenges, including COVID‐19 and Mpox. Yet, the systems that enabled these advances are fragile. As research funding decreases, the discovery pipeline risks slowing just as new long‐acting medications have demonstrated efficacy and transformative technologies like gene editing and immune‐based cures are within reach. Sustained commitment to HIV science is a catalytic investment in a healthier, more resilient world.

Scientific innovation, however, only matters if it is matched by accessible pricing, adequate supply and effective delivery models. Achieving this requires removing financial, legal and systemic barriers, and rethinking not only *what* health systems deliver, but *how* they deliver care. In the face of constrained resources, scaling up evidence‐based, cost‐effective delivery approaches is key, as are other innovations in efficient, effective and person‐centred modalities [5].

Domestic and regional investments in health systems are central to health sovereignty—the ability of countries to define, finance and sustain their health priorities independent of external actors, donors or geopolitical interests. Recent policy and funding shifts have exposed deep power imbalances and vulnerabilities in global health. Leadership of HIV programmes and research must be locally anchored, extending beyond ministries of health and national AIDS coordinating bodies to district‐level systems best placed to meet and respond to local needs. Decriminalizing stigmatized populations and behaviours and training providers to deliver person‐centred, stigma‐free care are central to creating enabling environments where evidence‐based interventions can be effectively implemented [6]. Domestic resource mobilization and innovative procurement mechanisms such as pooled procurement and social contracting are critical to advancing greater national self‐reliance in health financing and service delivery.

**Building rights‐based, person‐centred health systems through community leadership and accountability**

Communities are the engines of sustainability, equity and accountability in rights‐based health systems. As HIV programmes are further integrated into national financing, management and service delivery systems, communities must be equal partners in decision‐making as their leadership is essential to ensure evidence‐based, contextually relevant and equitable integration. Minimum community service packages can ensure that community priorities are reflected as countries redefine programmatic and investment priorities.

Community‐led and national monitoring systems are mutually reinforcing pillars of rights‐based, accountable health systems. Community‐led monitoring provides a powerful early warning system, driven by grassroots intelligence that can inform and assess the responsiveness and equity of health services. Reductions in funding have weakened these mechanisms and constrained civil society's ability to monitor service delivery and policy implementation. Strengthening such systems requires financial support that preserves independent oversight and community‐led principles. Strengthening national data systems is equally essential. Monitoring biomedical and non‐biomedical indicators is key to identifying barriers to service uptake, including social determinants of health. This requires systematic, routine data collection and public reporting of results at the most local levels, while respecting patient privacy. Just as important is ensuring that those data are accessible, transparent and routinely used by local decision‐makers, service providers and civil society.

**Equitable access to HIV prevention is key to epidemic control**

By the end of 2024, an estimated 3.8 million people globally had used oral pre‐exposure prophylaxis (PrEP) at least once during the year, representing progress but still far below the 2025 target of 10 million [7]. This gap highlights persistent barriers to access and uptake, even as innovation in HIV prevention accelerates.

The advent of long‐acting injectable PrEP (LA‐PrEP) offers a major opportunity to expand choice and coverage, particularly for people facing adherence or access challenges with daily oral PrEP. Yet, realizing this promise requires overcoming persistent financial and implementation barriers. Current pricing for LA‐PrEP is out of reach for most countries [8]. While recent agreements with generic manufacturers have improved the access outlook in some regions, large parts of the world, including much of Latin America and the Caribbean and Asia, remain left out of those deals [9, 10]. The implementation of LA‐PrEP creates new risks for HIV prevention, putting further strain on under‐staffed and ‐resourced clinics. Strengthening sub‐national public health systems where day‐to‐day resource allocation and delivery decisions are made is essential, as is investing in differentiated service delivery models that can improve access to high‐impact interventions in health facilities, communities and elsewhere [11, 12].

Digital approaches such as individual chat‐based support that links clients to appropriate clinical services and commodities (e.g. HIV self‐test kits, PrEP,  post‐exposure prophylaxis (PEP)) can offer a complementary pathway to relieve pressure on overstretched health systems. Mobile health and artificial intelligence (AI)‐based applications can tailor information, reminders and guidance to support sustained engagement and empower individuals to manage aspects of their health outside traditional clinical settings [13]. However, realizing the potential of these models requires strengthened evidence of long‐term effectiveness and robust privacy and data protection frameworks. When culturally grounded and integrated into national systems and strategies, digital tools can enhance engagement and equity.

Sustaining and scaling these prevention innovations demands stable financing. The abrupt reduction in U.S. funding for HIV programmes in 2025 resulted in widespread disruptions to treatment and prevention commodities and service delivery across multiple countries, potentially contributing to 10.75 million new HIV acquisitions and 2.9 million additional AIDS‐related deaths between 2025 and 2030 [14, 15]. Disruptions have been particularly acute for HIV prevention programming, impacting communities of key populations more severely. If not urgently addressed, these disruptions could reverse hard‐won gains.

The global HIV response demonstrates what is possible when science, policy and community leadership converge. As we head towards AIDS 2026, the principles outlined in the Kigali Declaration and the priorities emerging from the *Road to Rio* consultation provide a clear framework for action: invest in science, follow the evidence and build person‐centred health systems rooted in respect, dignity and trust. This means rethinking outdated models, rebuilding systems to put people first, and rising together through inclusive policies, innovation, and sustained collaboration. By aligning global advocacy around these priorities in concert with other health and justice movements, we can safeguard the gains of the past four decades and leverage them to shape integrated, resilient health systems for the future.

From Kigali to Rio and beyond, this is how we honour the HIV movement's legacy while advancing health for all. Now is the time to rethink, rebuild, and rise.

## COMPETING INTERESTS

The authors have no competing interests to declare.

## AUTHOR CONTRIBUTIONS

The concept for this viewpoint was developed by BG, RMO, SMA, RJ, AG and KN. All members of the Road to Rio Advisory Group contributed and approved the final version.

## ROAD TO RIO ADVISORY GROUP

Abdul‐Fatawu Salifu, Adriano Queiroz, Andrew Grulich, Andriy Klepikov, Angela Mushavi, Anna Bershteyn, Beatriz Grinsztejn, Brian Honermann, Charles B. Holmes, Edinah Mudimu, Imelda Mahaka, Kenneth Ngure, Laura Schaefli, Marlène Bras, Midnight Poonkasetwattana, Rena Janamnuaysook, Richard Muko, Sara M. Allinder, Valdiléa Veloso and Yvette Raphael.

## Data Availability

Data sharing not applicable to this article as no datasets were generated or analysed during the current study.
